# Biweekly oxaliplatin plus S1 for Chinese elderly patients with advanced gastric or gastroesophageal junction cancer as the first-line therapy: a single-arm, phase 2 study

**DOI:** 10.1186/s12885-022-09332-7

**Published:** 2022-03-09

**Authors:** Zhichao Jiang, Aiping Zhou, Yongkun Sun, Wen Zhang

**Affiliations:** Department of Medical Oncology, National Cancer Center/ National Clinical Research Center for Cancer/ Cancer Hospital, Chinese Academy of Medical Sciences and Peking Union Medical College. No, 17, Panjiayuannanli Street, Chaoyang District Beijing, 100021 China

**Keywords:** Oxaliplatin, S1, Objective response rate, Advanced G/GEJ adenocarcinoma

## Abstract

**Background:**

SOX (oxaliplatin and S1, every 3 weeks) is one of the most common first-line chemotherapy for advanced or metastatic G/GEJ (gastric or gastroesophageal junction) cancer in Asia, but it has noticeable hematological and neurological toxicity. In China, the majority of gastric cancer patients are middle-aged and elderly with poor tolerance to 3-weekly chemotherapy. Therefore, we aimed to assess efficacy and safety of biweekly SOX for Chinese advanced G/GEJ cancer patients aged ≥ 60 years as the first-line treatment in a single arm phase 2 study.

**Methods:**

Oxaliplatin was administered intravenously on day 1 at 85 mg/m^2^. S-1 was given at 80, 100 or 120 mg/day, depending on the body surface area (< 1.25 m^2^, 1.25 to < 1.5 m^2^, or ≥ 1.5 m^2^), twice daily, on day 1–10, every 2 weeks. The primary endpoint was objective response rate (ORR), and the secondary endpoints included progression free survival (PFS), overall survival (OS), disease control rate (DCR), duration of response (DOR) and safety.

**Results:**

Between May 2016 and Sep 2018, 42 patients were enrolled. The median follow-up was 43.6 months. The ORR and DCR were 52.4% and 85.7%, respectively. The median PFS was 4.6 months (95%CI 2.486–6.714), and the median OS was 11.1 months (95%CI 8.001–14.199). The most common treatment-related adverse events (TRAEs) of any grade included thrombocytopenia (59.5%), neutropenia (57.1%), appetite loss (57.1%) and nausea (54.8%). Only two patients suffered from grade 3 TRAEs (4.8%), including neutropenia (1 patient, [2.4%]) and diarrhea (1 patient, [2.4%]). No ≥ grade 4 TRAEs occurred.

**Conclusions:**

Biweekly SOX seemed to have favorable tolerance without compromising the efficacy as the first-line therapy in Chinese elderly patients aged ≥ 60 years with advanced G/GEJ cancer.

**Trial registration:**

ClinicalTrials.gov ID: NCT04694404 (5/1/2021). This study was approved by the Ethical Committee of National Cancer Center/ National Clinical Research Center for Cancer/ Cancer Hospital, Chinese Academy of Medical Sciences and Peking Union Medical College, (17–048/1303).

## Background

Gastric cancer is the second most common cancer and the third leading cause of cancer related deaths in China [1, 2]. The incidence of gastric cancer is 63.92 per 100, 000 among the population aged 50–59 years, and 143.10 per 100, 000 among those aged 60–69 years, and the morbidity increases with age [3]. The majority of Chinese gastric cancer patients are middle-aged and elderly. Considering the population characteristics, medical services and social economy of different parts of China, people over the age of 60 years old are defined as the elderly in China[4]. These Chinese elderly patients usually have a poor tolerance and compliance to chemotherapy due to the declination of organ functions or the existing physical illnesses, such as diabetes, hypertension and other cardiac and cerebral diseases. Suspension or discontinuation of chemotherapy may decrease the objective response rate (ORR) or survival. Therefore, it is necessary to optimize the chemotherapy regimen for elderly advanced gastric cancer patients aged ≥60 years to improve the tolerance and efficacy of chemotherapy in China.

SOX (oxaliplatin and S1, every 3 weeks) is one of the first-line treatments for advanced gastric cancer patients, which is recommended by the guidelines of Chinese Society of Clinical Oncology (CSCO) and Japanese Gastric Cancer Association (JGCA). Nevertheless, 3-weekly SOX regimen is usually related to a high incidence of neutropenia and thrombocytopenia, which is reported to be 56.9–82.5% and 44.7–84.1% respectively [5–10]. Due to the noticeable hematologic toxicity, dose reduction is preferred by more patients. The proportion is reported to be 36.1–41.5% and 41.4–48.5% for S1 and oxaliplatin, respectively, in previous phase II and III clinical trials [5–7]. Therefore, the efficacy and safety of modified 3-weekly or biweekly chemotherapy regimens with oxaliplatin and S1 or capecitabine have been evaluated in a series of clinical studies. Biweekly XELOX (oxaliplatin 85 mg/^2^ at day 1, capecitabine 900-1800 mg/m^2^ bid at day 1 to 10, every 2 weeks) was demonstrated to be related to a better tolerance and satisfactory therapeutic outcomes for the untreated advanced colorectal cancer and gastric cancer patients, especially the elderly patients [11, 12]. Furthermore, compared with the advanced gastric cancer patients with a median age of 76 years who accepted standard 3-weekly XELOX (HR 1.10, 95% CI, 0.90–1.33), the counterparts who received modified 3-weekly XELOX with dose reduction (HR 1.09, 95% CI, 0.89–1.32) could achieve a similar PFS benefit [13]. The patients who took 60% of the standard dose showed a higher satisfaction in the quality of life and objective response rate (43% vs. 35%) [13]. Moreover, metronomic chemotherapy was related to a smaller toxicity and satisfactory survival benefit in lung cancer and breast cancer patients by reducing single dose or shortening the intermission of chemotherapy. Thus, we evaluated the efficacy and safety of biweekly SOX (oxaliplatin and S1) for advanced or metastatic elderly gastric cancer patients as the first-line treatment, to optimize the chemotherapy for the Chinese elderly patients with poor physical status.

## Methods

### Study design and objective

This was a single-arm, prospective phase II study which aimed to evaluate the efficacy and safety of biweekly SOX (oxaliplatin plus S1 every two weeks) for Chinese advanced G/GEJ cancer patients aged ≥ 60 years old as the first-line treatment.

### Patients

Inclusion criteria: 1) aged ≥ 60 years old; 2) histological or cytological diagnosis of G/GEJ (gastric or gastroesophageal junction) adenocarcinoma, no matter the status of HER2; 3) untreated, unresectable advanced or metastatic disease (patients who had tumor progression at least 12 months after adjuvant or neoadjuvant chemotherapy were also eligible); 4) at least one measurable lesion according to Response Evaluation Criteria in Solid Tumors (RECIST, version 1.1); 5) Eastern Cooperative Oncology Group (ECOG) performance status of ≤ 2; 6) acceptable hematological, hepatic, and renal function with a predicted life expectancy of at least 3 months. Exclusion criteria: 1) diagnosis of other malignant tumors (except cured basal cell carcinoma of skin and squamous cell carcinoma of skin, or cervical carcinoma in situ) within 5 years; 2) the total dose of oxaliplatin within 2 years ≥ 800 mg/m^2^ in the adjuvant or neoadjuvant chemotherapy; 3) allergic to oxaliplatin or S-1.

### Procedures

S-1 was given orally twice daily during the first 10 days of a 2-week cycle. The dose was 80 mg/day for the body surface area (BSA) < 1.25 m^2^, 100 mg/day for BSA ≥ 1.25 to < 1.5 m^2^, and 120 mg/day for BSA ≥ 1.5 m^2^. Oxaliplatin was given intravenously at a dose of 85 mg/m^2^ on day 1 of each 2-week cycle. The treatment was continued until disease progression (defined according to RECIST, version 1.1), unacceptable toxicity, or patient withdrawal, whichever occurred first. For the patients with tumor response, the oxaliplatin-based doublet chemotherapy was given for a maximum of 9 cycles, and then S-1 maintenance chemotherapy was prescribed.

Adverse events were assessed by Common Toxicity Criteria version for Adverse Events, version 4.0. The dose of S-1 was reduced to the next level, if grade 4 hematological toxicities, ≥ grade 3 febrile neutropenia/leukopenia, gastrointestinal toxicities or stomatitis, or Cr > 132.6 μmol/L was found. If ≥ grade 2 peripheral neurotoxicity or any ≥ grade 3 toxicity continued for 7 days, the dose of oxaliplatin was reduced from 85 mg/m^2^ to 70 mg/m^2^ and 60 mg/m^2^ based on the schedule.

Tumor response was evaluated every 6 weeks according to RECIST version 1.1. Contrast enhanced computed tomography (CT) or magnetic resonance imaging (MRI) was recommended.

The primary endpoint was ORR, which was defined as the percentage of the patients who achieved complete or partial response. The secondary endpoints were progression free survival (PFS, defined as the time from the initiation of chemotherapy to the first progression or death of any cause), overall survival (OS, defined as the time from the initiation of chemotherapy to death of any cause), disease control rate (DCR), duration of response (DOR) and safety.

The protocol was approved by the Ethical Committee of National Cancer Center/ National Clinical Research Center for Cancer/ Cancer Hospital, Chinese Academy of Medical Sciences and Peking Union Medical College, (17–048/1303). Clinical trial information.: NCT04694404 (5/1/2021).

### Statistical analysis

The ORR of S1 was about 30%, and the expected ORR in the current study was 50% because the treatment was combined with oxaliplatin. Based on the assumption of a one-tailed score test with an α of 0.05, at least 40 patients were required to be included to ensure the statistical power of ≥ 80%. Considering a drop-out rate of 10%, 44 patients were required to be included finally. All statistical analyses were performed using SPSS (version 25). Kaplan–Meier method was used to analyze the median PFS and OS.

## Results

### Patient characteristics

Between May 2016 and Sep 2018, 42 eligible patients received the treatment at the Department of Medicine Oncology, National Cancer Center/ National Clinical Research Center for Cancer/ Cancer Hospital. As shown in Table 1, the median age of the patients was 68 years (range, 60–79 years), and ECOG was 0 in 25 patients (59.5%) and 1 in 17 patients (40.5%). The most common underlying physical illnesses included hypertension (21.4%), diabetes (11.9%), coronary heart disease (11.9%) and cerebral infarction (7.1%).

**Table 1:** The baseline characteristics of the patients

	No	%
Gender		
Male	34	81.0
Female	8	19.0
Age (years)		
60 ~ < 65	9	21.4
65 ~ < 70	18	42.9
70 ~ < 75	11	26.2
≥ 75	4	9.5
ECOG		
0	25	59.5
1	17	40.5
BMI		
Low (< 18.5 kg/m^2^)	5	11.9
Normal (18.5 ~ < 24 kg/m^2^)	22	52.4
Overweight (24 ~ < 27 kg/m^2^)	10	23.8
Obesity (27 ~ < 30 kg/m^2^)	3	7.1
Severe obesity (≥ 30 kg/m^2^)	1	2.4
Unknown	1	2.4
Disease status		
Recurrent	4	9.5
Newly diagnosed	38	90.5
HER2 status		
HER2 positive	7	16.7
HER2 negative	30	71.4
Unknown	5	11.9
Metastatic site		
Retroperitoneal lymph nodes	25	59.3
Supraclavicular lymph nodes	16	38.1
Mediastinal lymph nodes	12	28.6
Liver	10	23.8
Peritoneal	10	23.8
Lung	10	23.8
Adrenal gland	2	4.8
Bone	1	2.4
Ovary	1	2.4
Existing physical illness		
Hypertension	9	21.4
Diabetes	5	11.9
Coronary heart disease	5	11.9
Cerebral infarction	3	7.1
Carotid artery stenosis	2	4.8
Arrhythmia	2	4.8
Liver cirrhosis	1	2.4

### Response and survival

**Fig. 1:**
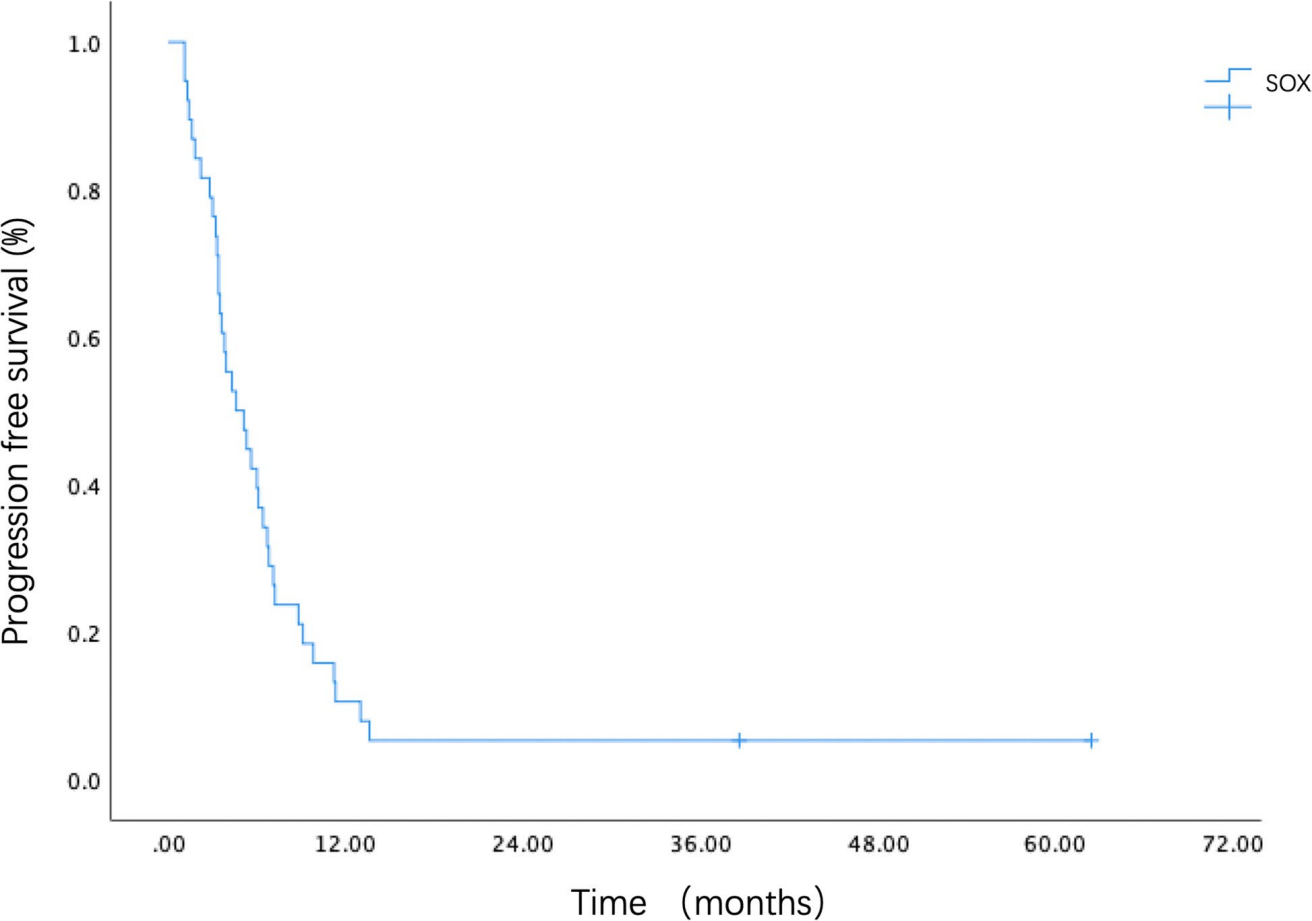
Kaplan–Meier estimates of PFS of advanced G/GEJ cancer patients treated with biweekly SOX. PFS, progression free survival; G/GEJ cancer, gastric or gastroesophageal junction caner

**Fig. 2:**
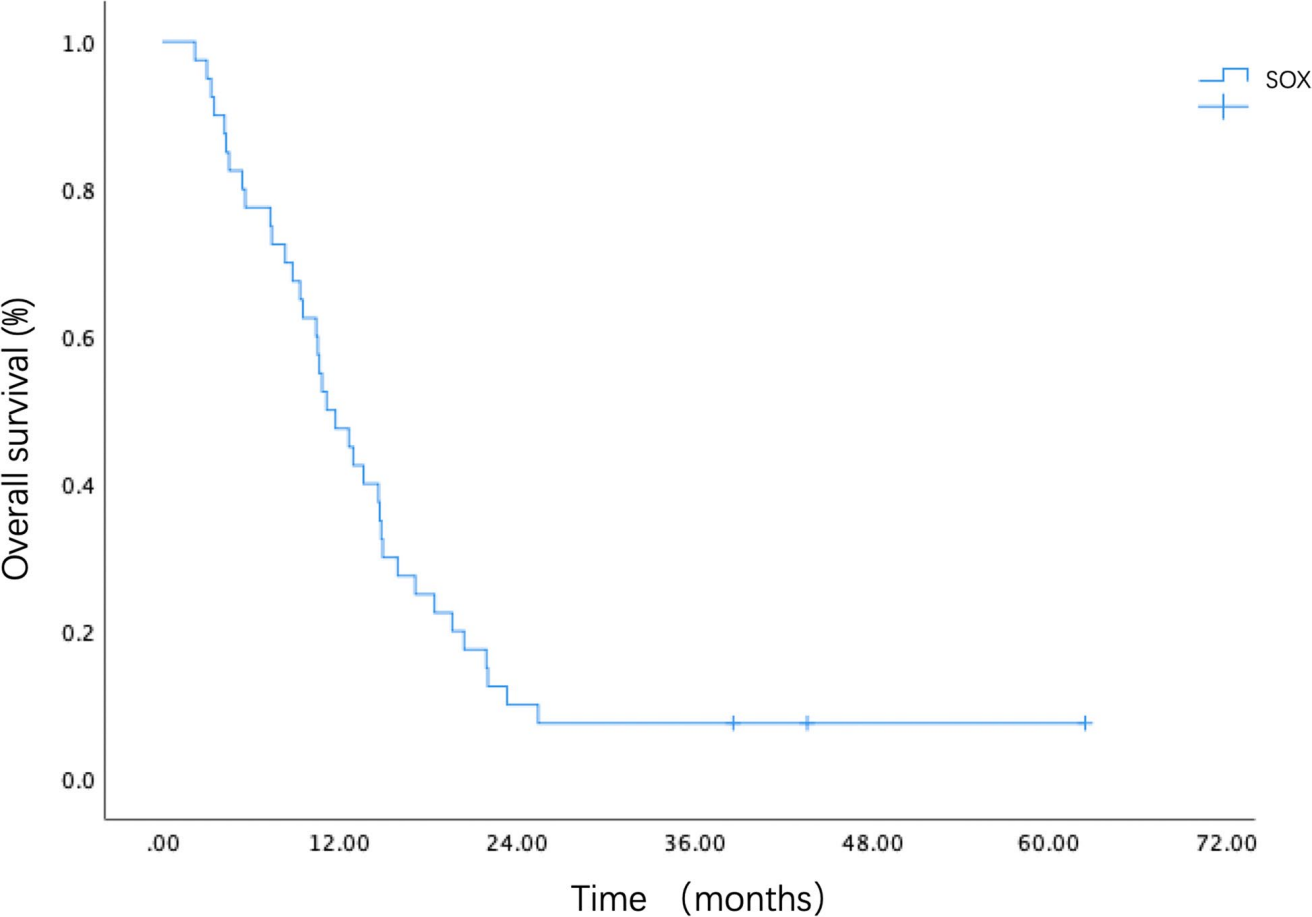
Kaplan–Meier estimates of OS of advanced G/GEJ cancer patients treated with biweekly SOX. OS, overall survival; G/GEJ, gastric or gastroesophageal junction

Objective response was observed in 22 (52.4%) of 42 patients. Stable disease was achieved in 14 patients (33.3%), and 6 patients (14.3%) suffered disease progression during the treatment. The assessed DCR was 85.7% (36/42). Among the 22 patients who achieved an objective response, the median time to response was only 1.5 months, and the duration of response was up to 4.6 months (95%CI 2.245–6.955). The best overall response is shown in Table 2. During the median follow-up of 43.6 months, the median PFS was 4.6 months (95%CI 2.486–6.714, Fig. 1), and the median OS was 11.1 months (95%CI 8.001–14.199, Fig. 2). Retrospective analysis showed the patients with low body mass index (BMI) (< 18.5 kg/m^2^) had a shorter OS of 7.4 months, while the patients with normal BMI (18.5 to 24 kg/m^2^), overweight (BMI 24-27 kg/m^2^) or obesity (BMI 27-30 kg/m^2^) had a median OS of 10.6 months, 12.6 months and 11.1 months, respectively (*p* = 0.188). Nevertheless, the severe obese patients with BMI over 30 kg/m^2^ only had a survival of 4.2 months. Moreover, the therapeutic outcomes seemed better in patients with ECOG performance status of 0. The median OS of the patients with ECOG performance status of 0 and 1 was 12.9 months and 10.6 months, respectively (*p* = 0.719). In addition, the ORR of the patients < 70 and ≥ 70 years old were 44.4% and 66.7% (*p* = 0.167). The median PFS were 3.8 and 6.3 months (*p* = 0.078), respectively. There was no statistical difference of efficacy endpoints between the patients < 70 and ≥ 70 years old.

**Table 2:** The best overall response (*N* = 42) in patients treated with biweekly SOX

The best response (*N* = 42)	No. (%)
CR	0 (0%)
PR	22 (52.5%)
SD	14 (33.3%)
PD	6 (14.3%)
ORR (CR + PR)	22 (52.5%)
DCR (CR + PR + SD)	36 (85.7%)

*CR* Complete response, *PR* Partial response, *SD* Stable disease, *PD* Progressive disease, *ORR* Objective response rate, *DCR* Disease control rate

### Tolerability and Adverse Events

The common TRAEs of any grade included thrombocytopenia, neutropenia, gastrointestinal reaction, hyperbilirubinemia and fatigue. Most of these adverse events (AEs) were reported to be grade 1–2. The most common grade 3 TRAEs included neutropenia (*n* = 1, 2.4%) and diarrhea (*n* = 1, 2.4%). No febrile neutropenia and other grade 3 or worse AEs were found. The adverse event profiles are presented in Table 3. The median number of treatment cycles was 6, and 26 patients (61.9%) completed at least 6 cycles of chemotherapy. No TRAEs led to study discontinuation or interruption. Dose was reduced in 2 patients (4.8%) because of grade 2 thrombocytopenia with a long recovery time.

**Table 3:** Adverse events of the patients after treatment

Adverse events	Any		Grade 1		Grade 2		≥ Grade 3	
	No	(%)	No	(%)	No	(%)	No	(%)
Appetite loss	24	57.1	19	45.2	5	11.9	0	0
Nausea	23	54.8	19	45.2	4	9.5	0	0
Vomiting	12	28.6	7	16.7	5	11.9	0	0
Diarrhea	4	9.5	3	7.1	0	0	1	2.4
Neutropenia	24	57.1	16	38.1	7	16.7	1	2.4
Thrombocytopenia	25	59.5	12	28.6	13	31.0	0	0
Increased ALT	9	21.4	9	21.4	0	0	0	0
Increased AST	12	28.6	12	28.6	0	0	0	0
Hyperbilirubinemia	16	38.1	16	38.1	0	0	0	0
Rash	1	2.4	0	0	1	2.4	0	0
Fatigue	13	31.0	9	21.4	4	9.5	0	0
Weight loss	8	19.0	5	11.9	3	7.1	0	0
Peripheral neuropathy	8	19.0	7	16.7	1	2.4	0	0

### Second-line treatment

Until the last follow-up, disease progression was confirmed in 38 patients. 24 patients (65%) accepted the second-line anti-tumor treatments, including chemotherapy (*n* = 20), local radiotherapy (*n* = 1) and clinical trial therapy (*n* = 3). Chemotherapy regimens included taxanes plus raltitrexed (*n* = 7), apatinib (*n* = 5), taxanes (*n* = 3), irinotecan plus apatinib (*n* = 1), raltitrexed (*n* = 1), oxaliplatin plus raltitrexed (*n* = 1), docetaxel plus S1 (*n* = 1) and unknown (*n* = 1). Further treatment was refused by four patients after disease progression. Whether the other ten patients received subsequent treatment was not clear.

## Discussion

Platinum-based drug plus fluorouracil is recognized as the standard first-line chemotherapy for metastatic gastric cancer. S1 is a new oral drug of fluorouracil, which combines tegafur with two modulators of gimeracil and oteracil [14], and is related to a lower incidence of hand foot syndrome (HFS) compared with capecitabine. S1 plus platinum has favorable efficacy for Asian advanced gastric cancer patients. ORR and DCR of SOX were reported to be 32.6–58% and 75–85.2%, respectively, in the randomized phase III studies. The median PFS and OS were 5.5–5.7 months and 12.9–14.1 months, respectively [5, 6, 8]. Especially for the non-intestinal Lauren’s type gastric cancer, SOX was related to a prolonged OS compared with SP in Chinese patients [5]. Given an excellent efficacy and acceptable toxicity [5, 6, 15, 16], SOX is one of the most common regimens for the first-line chemotherapy of advanced gastric cancer in Asia. However, Chinese elderly gastric cancer patients usually have poor performance status and underlying diseases, such as hypertension, diabetes and coronary heart disease. Therefore, S1 monotherapy was a choice for the patients who were intolerant to 3-weekly combination chemotherapy. Nevertheless, the efficacy of S1 monotherapy as the first-line treatment was limited. The ORR was only 24.7–31%, and the median OS was 10.5–10.8 months [17–19], which was worse compared to the chemotherapy of fluoropyrimidine and platinum [10]. Therefore, it is urgent to explore a therapy with satisfactory efficacy and acceptable tolerability for Chinese elderly patients of advanced gastric cancer.

This phase 2 study preliminarily indicated that biweekly SOX was related to a favorable survival as the first-line therapy in Chinese elderly advanced gastric cancer patients with a median age of 68 years old. Compared with S1 monotherapy, biweekly SOX improved the efficacy with a higher ORR (54.2%) and DCR (85.7%). And the median PFS and OS of the patients treated by biweekly SOX were 4.6 months and 11.1 months, respectively, which were similar to those of the patients treated by 3-weekly SOX. And both the patients < 70 and ≥ 70 years old could acquire similar survival benefit from biweekly SOX in our study. Furthermore, in another phase II study, the therapeutic outcomes of biweekly SOX (oxaliplatin 85 mg/m^2^ d1, S1 80-120 mg/day d1-7, every 2 weeks) as the first-line treatment were evaluated in Chinese gastric cancer patients. The ORR and DCR were 30.43% and 76.08%, respectively [20]. The patients with a median age of 59 years old had a PFS of 4.4 months and OS of 10.3 months [20], who were much younger than our patients. In that study, 78.3% of the patients accepted the second-line chemotherapy, whereas the proportion of our patients was 63.2%. In our study, 7 HER2 positive patients didn’t accept trastuzumab for financial reasons, which might have a negative effect on the survival. However, biweekly SOX in our study showed similar therapeutic outcomes for the Chinese elderly patients compared with the counterpart in the previous phase II study.

A series of clinical studies demonstrated that 3-weekly SOX (oxaliplatin 130 mg/m^2^ d1, S1 80-120 mg/day d1-7, every 3 weeks) was related to noticeable hematological and neurological toxicity [7–10]. Therefore, the dose of oxaliplatin was reduced to 100 mg/m^2^ every three weeks in the G-SOX phase 3 study. However, the modified 3-weekly SOX didn’t show lower toxicity. The incidence of grade 3–4 neutropenia and thrombocytopenia was still reported to 19.5% and 10.1%, respectively. The incidence of any grade peripheral neuropathy was up to 85.5%. As a result, the dose of oxaliplatin was reduced in 48.5% of the patients, and the treatment was discontinued in 5% of the patients in the G-SOX phase 3 study [5]. Advanced gastric cancer patients usually suffered from weight loss, malnutrition and chronic anemia, which might impair the tolerance to anti-tumor therapy, especially for the elderly patients. In our study, about 1/4 of the patients had cardiovascular or cerebrovascular disease, with a high-risk acute attack of the underlying diseases if severe TRAEs occurred. This study showed good tolerance of biweekly SOX by reducing single dose of oxaliplatin and shortening the treatment course of S1 in these elderly patients. The incidence of any grade neutropenia was 57.1%, which was similar to the result reported by a previous biweekly SOX phase 2 study (54.35%) [20], and lower than 65.6% and 68.9% in two 3-weekly SOX phase 3 studies [5, 6]. Only 2.4% patients developed grade 3 neutropenia, and no grade 4 neutropenia occurred. There was no ≥ grade 3 thrombocytopenia observed in this study, while the incidence was up to 7.5–17% for 3-weekly SOX [5–10]. Furthermore, this biweekly SOX was related to a better tolerance to peripheral neurotoxicity and digestive symptoms compared with 3-weekly SOX, and any grade peripheral neuropathy, nausea and vomiting were less common (19% vs. 34–85.5%, 54.8% vs. 61.5–74.6% and 28.6% vs. 34.9 ~ 59.9%) [5–10]. Most of these elderly patients showed good physical conditions during treatment. As a result, biweekly SOX might bring a better quality of life to the Chinese elderly advanced gastric cancer patients with lower toxicity.

Moreover, the patients with better nutritional and physical status seemed to have longer OS in this study. Compared with those with lower BMI, the patients with BMI ≥ 18.5 kg/m^2^ showed a longer OS of 3.2–5.2 months numerically (*p* = 0.188). The OS of the patients with ECOG of 0 and 1 was 12.9 months and 10.6 months, respectively (*p* = 0.719). Nutritional and physical status was closely correlated with immune function and treatment tolerance [21, 22], which might compromise the therapeutic outcomes. Elderly patients usually had a lower level of albumin and lymphocytes, which was associated with poorer nutritional status and immune function [23–26]. It was found that nutritional guidance and intervention during chemotherapy caused an increase in the CD4 + lymphocytes and alleviated the hematological and digestive toxicity [27]. Therefore, integration of nutritional supportive care into chemotherapy might be helpful to improve the tolerance of elderly patients to anti-tumor treatment, and subsequently prolong the survival.

This study also has limitations. First, it was not randomized. Second, the sample size was small. Third, the trial was carried out in a single institution. Therefore, our preliminary findings remain to be further verified by well-designed randomized studies with a larger sample size. Besides, considering the population characteristics, the Chinese elderly patients were defined as over the age of 60 years old. It might be more suitable for Chinese national conditions. While, according to the WHO standard, these patients belonged to the young elderly patients.

## Conclusions

Biweekly SOX seemed to have favorable tolerance without compromising the efficacy as the first-line therapy in Chinese elderly patients aged ≥ 60 years with advanced G/GEJ cancer. The incidence of grade 3 or worse AEs was low.

## Data Availability

The datasets generated during and analyzed during the current study are not publicly available due to patients’ confidentiality but are available from the corresponding author on reasonable request.

## Abbreviations

G/GEJ: Gastric or gastroesophageal junction
ORR: Objective response rate
PFS: Progression free survival
OS: Overall survival
DCR: Disease control rate
DOR: Duration of response
CSCO: Chinese Society of Clinical Oncology
JGCA: Japanese Gastric Cancer Association
ECOG: Eastern Cooperative Oncology Group
BSA: Body surface area
TRAEs: Common treatment-related
AE: Adverse events adverse events
HFS: Hand foot syndrome
BMI: Body mass index
